# Supplementary material for: The Role of Opioid Receptor Antagonists in Regulation of Blood Pressure and T-Cell Activation in Mice Selected for High Analgesia Induced by Swim Stress
Source: Int J Mol Sci. 2024 Feb 23;25(5):2618. doi: 10.3390/ijms25052618 (PMC10932203; doi:10.3390/ijms25052618)
Supplement: Supplementary file 1 [file ijms-25-02618-s001.zip › ijms-2853416-supplementary.pdf]

Table S1. List of antibodies.

SPECIFICITY	FLUOROCHROME	CLONE NAME	SUPPLIER
**CD3E**	PerCP	145-2C11	BioLegend
**CD8A**	AF700	53-6.7	BioLegend
**CD4**	BV750	GK 4.5	BioLegend
**CD69**	PEC7	H1.2F3	BioLegend
**CD44**	AF647	IM7	BioLegend
**CD62L**	PE594	MEL-14	BioLegend

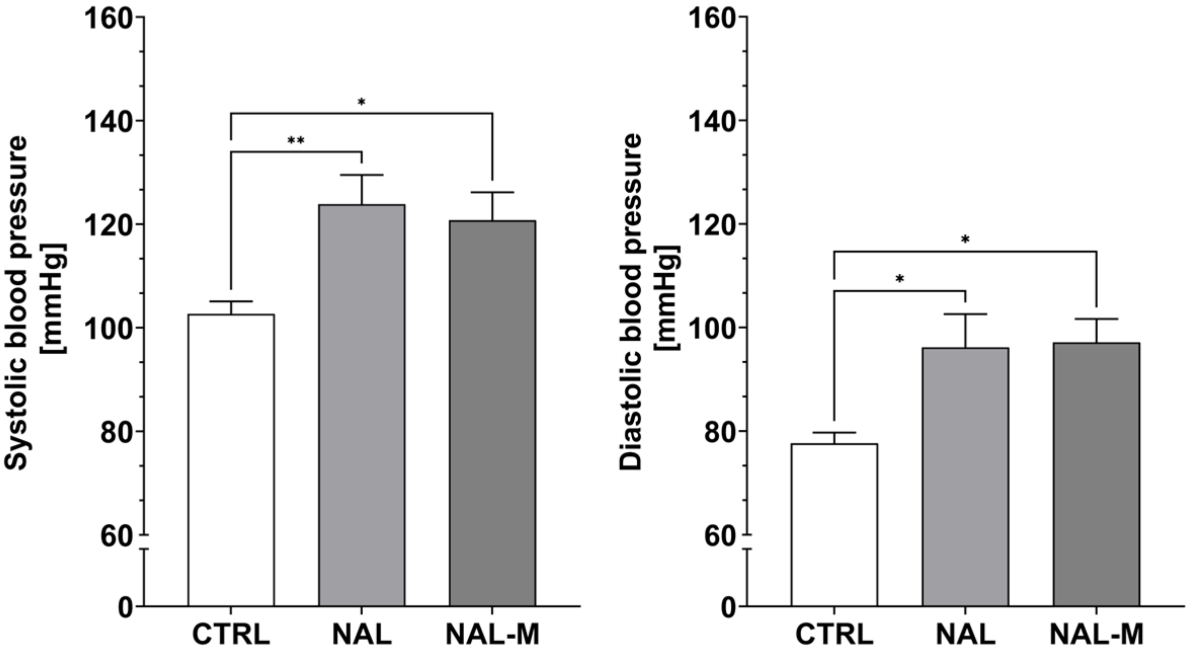

Figure S1. Mean systolic and diastolic blood pressure after non-selective opioid system antagonism during 10 days of treatment. One or two symbols represent post-hoc comparisons $p < 0.05$, $p < 0.01$, respectively. Abbreviations: CTRL – control group; NAL – naloxone; NAL-M – naloxone methiodide.

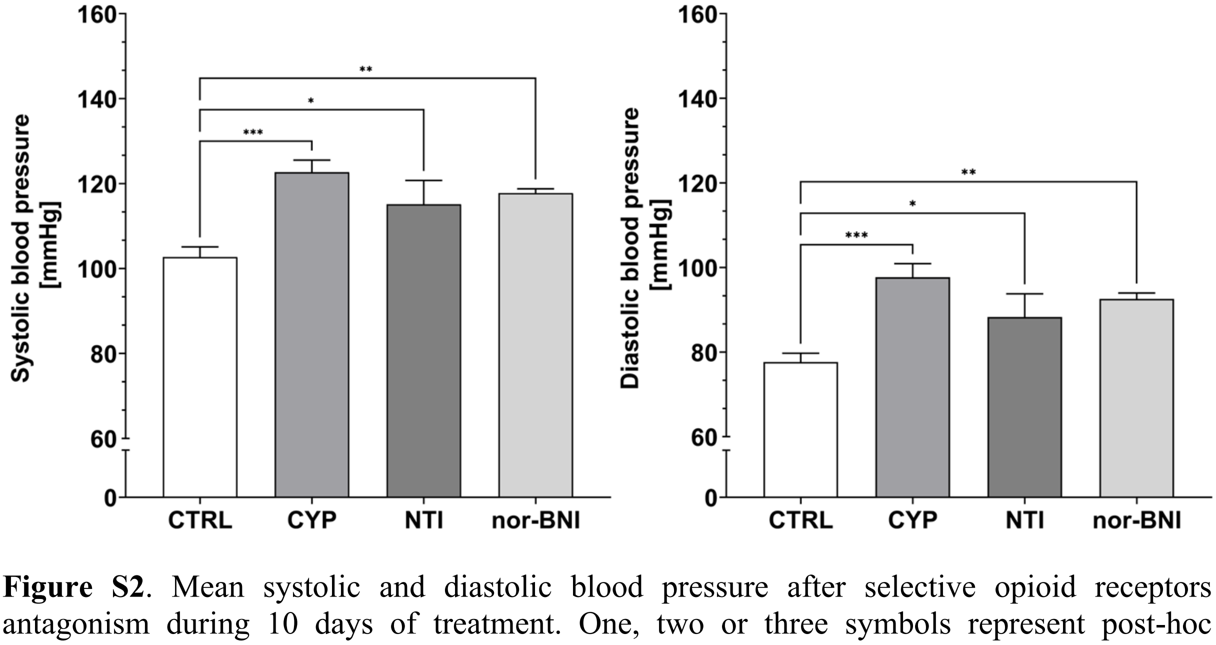

Figure S2. Mean systolic and diastolic blood pressure after selective opioid receptors antagonism during 10 days of treatment. One, two or three symbols represent post-hoc comparisons $p < 0.05$, $p < 0.01$, $p < 0.001$, respectively. Abbreviations: CTRL – control group; CYP – cyprodime hydrochloride; NTI – naltrindole hydrochloride; nor-BNI – nor-binaltorphimine dihydrochloride.
